# Fetal metabolic adaptations to cardiovascular stress in twin-twin transfusion syndrome

**DOI:** 10.1016/j.isci.2023.107424

**Published:** 2023-07-20

**Authors:** Jacqueline G. Parchem, Huihui Fan, Lovepreet K. Mann, Qiuying Chen, Jong H. Won, Steven S. Gross, Zhongming Zhao, Heinrich Taegtmeyer, Ramesha Papanna

**Affiliations:** 1 Department of Obstetrics, Gynecology & Reproductive Sciences, Division of Maternal-Fetal Medicine, McGovern Medical School, The University of Texas Health Science Center at Houston, Houston, TX, USA; 2 Center for Precision Health, School of Biomedical Informatics, The University of Texas Health Science Center at Houston, Houston, TX, USA; 3 Department of Neurology, McGovern Medical School, The University of Texas Health Science Center at Houston, Houston, TX, USA; 4 The Fetal Center at Children’s Memorial Hermann Hospital, Houston, TX, USA; 5 Department of Pharmacology, Weill Cornell Medicine, New York, NY, USA; 6 Department of Internal Medicine, Division of Cardiology, McGovern Medical School, The University of Texas Health Science Center at Houston, Houston, TX, USA

**Keywords:** Health sciences, Developmental anatomy, Cardiovascular medicine, Biology of human development

## Abstract

Monochorionic-diamniotic twin pregnancies are susceptible to unique complications arising from a single placenta shared by two fetuses. Twin-twin transfusion syndrome (TTTS) is a constellation of disturbances caused by unequal blood flow within the shared placenta giving rise to a major hemodynamic imbalance between the twins. Here, we applied TTTS as a model to uncover fetal metabolic adaptations to cardiovascular stress. We compared untargeted metabolomic analyses of amniotic fluid samples from severe TTTS cases vs. singleton controls. Amniotic fluid metabolites demonstrated alterations in fatty acid, glucose, and steroid hormone metabolism in TTTS. Among TTTS cases, unsupervised principal component analysis revealed two distinct clusters of disease defined by levels of glucose metabolites, amino acids, urea, and redox status. Our results suggest that the human fetal heart can adapt to hemodynamic stress by modulating its glucose metabolism and identify potential differences in the ability of individual fetuses to respond to cardiovascular stress.

## Highlights

• TTTS is a unique model to examine fetal metabolic adaptations to cardiovascular stress
• Amniotic fluid metabolites reveal significant changes in energy metabolism in TTTS
• Identification of distinct metabolic clusters underscores the heterogeneity of TTTS
• Our study highlights potential differences in individual fetal responses to stress

## Graphical abstract

**Figure:**
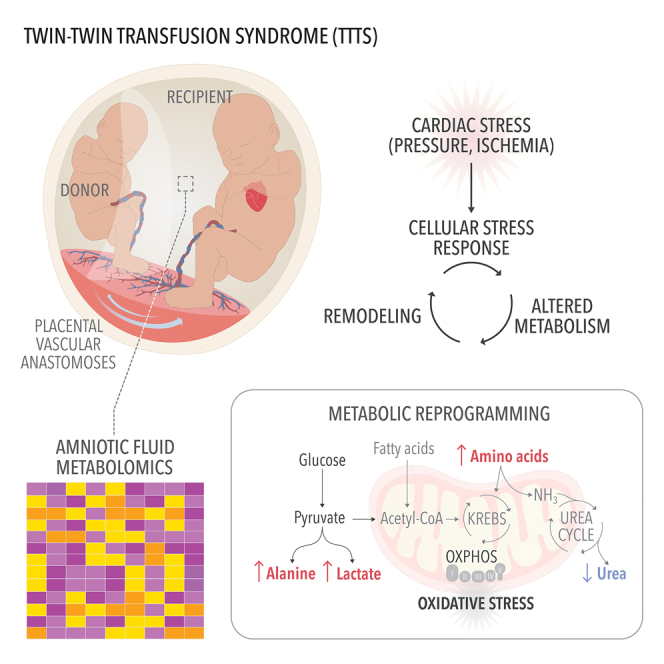

## Introduction

Monochorionic-diamniotic twin pregnancies comprise 70% of identical or monozygotic twins and are susceptible to unique complications arising from a single placenta shared by two fetuses (monochorionicity), each within its own amniotic sac (diamnionicity). Fetal health is intertwined by vascular connections within the placenta which serve as conduits for bidirectional blood flow and control the hemodynamic equilibrium between the twins. With a significant imbalance in blood flow from one fetus to the other, the twins are at risk for a complication termed twin-twin transfusion syndrome (TTTS). In this syndrome, one fetus becomes hypovolemic (the donor), while the other becomes volume overloaded (the recipient). The massive volume shift to the recipient leads to hypoperfusion of the donor’s kidneys, activation of the renin-angiotensin-aldosterone system (RAAS), and circulation of vasoactive mediators, promoting a vicious cycle.^1^^,^^2^ In severe cases of TTTS the outcome is dramatic. Indeed, both fetuses may experience extreme cardiovascular stress and cardiac failure, but for different causes. Left untreated, the fetal death rate approaches 90%, and one-half of survivors are neurodevelopmentally impaired.^3^

TTTS complicates approximately 10% of monochorionic-diamniotic twin pregnancies.^4^ Although TTTS affects a relatively small proportion of all pregnancies, the condition offers a unique opportunity to interrogate fetal physiology and cardiovascular adaptation.^5^ How the fetus responds to cardiovascular stress is of broad interest given the high prevalence of conditions that affect cardiovascular function *in utero* (e.g., maternal diabetes, infection, genetic conditions, and fetal anomalies).^6^ Surgical treatment of TTTS involves laser ablation of the offending placental vessels during the second trimester of pregnancy, which enables the procurement of amniotic fluid for molecular analysis during a window of fetal adaptation to cardiovascular stress—information that would otherwise not be available during pregnancy. Indeed, the amniotic fluid cell-free transcriptome reflects gene expression from many organs, including the developing heart.^7^^,^^8^ Furthermore, recent TTTS amniotic fluid microRNA (miRNA) studies found enrichment of miRNAs with roles in the cardiomyocyte stress response and metabolism^9^ and reported differences in amniotic fluid miRNAs of TTTS recipients with severe cardiomyopathy compared with to those with preserved cardiac function.^10^ These untargeted global molecular analyses reveal the utility of amniotic fluid for monitoring fetal health and are essential for hypothesis generation, identification of prognostic biomarkers, and furthering our knowledge of conditions such as TTTS that cannot be effectively modeled in animals.^11^

Variability in the natural course of TTTS raises the question of why some twins progress very rapidly to severe disease with decompensated heart failure, while others exhibit only changes in amniotic fluid volume, a proxy for fetal urine output. Although variation in the anatomy of the placental vasculature provides a mechanical explanation for TTTS, differences in fetal susceptibility to hemodynamic stress offers a potential contributing physiologic explanation. Determining the molecular underpinnings of the fetal response is critical for understanding short- and long-term effects on a child’s health, particularly when considering the impact on fetal programming, also known as the Developmental Origins of Health and Disease (DOHaD) hypothesis. Fetal programming is the concept that events or insults occurring during critical points of fetal development have long-lasting effects on the individual, especially with respect to future cardiovascular and metabolic disease risk.^12^ Many mechanisms of fetal programming have already been proposed, including epigenetic modifications, effects on gene expression, and disturbances in fetal hypothalamic-pituitary-adrenal axis development.^13^^,^^14^

At the same time, cell plasticity and proliferation in fetal life are required for rapid growth and development of the organism and adaptation to its environment. Notably, in contrast to the adult heart, fetal cardiomyocytes are capable of substantial regeneration.^15^ This developmental flexibility appears to endow the fetus with the ability to withstand and recover from significant hemodynamic stress.^16^^,^^17^ Indeed, we have proposed that reversion to the fetal genetic and metabolic programs is a protective mechanism and hallmark of adult heart failure.^18^ Reconciling the predicted permanent consequences of fetal programming with the inherent flexibility of the developing fetus remains an unresolved issue of far-reaching clinical relevance. Here we explored the fetal response to cardiovascular stress using untargeted metabolite profiling of amniotic fluid from fetuses with severe TTTS to gain further insights into possible mechanisms and found heterogeneity among fetuses with severe TTTS.

## Results

### Clinical characteristics of TTTS and control pregnancies

**Figure 1:**
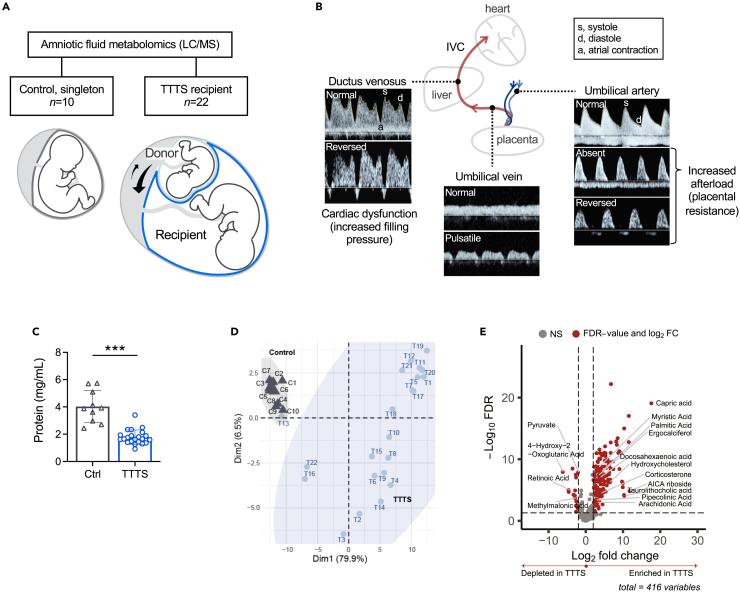
Untargeted amniotic fluid metabolomics identifies metabolic changes in TTTS fetuses with cardiovascular stress (A) Overview of study groups. (B) Stage III TTTS is defined by abnormal Doppler studies of the umbilical artery, ductus venosus, or umbilical vein. Representative images of normal and abnormal Doppler patterns from TTTS cases included in the study are shown. IVC, inferior vena cava. (C) Dilution of amniotic fluid protein in TTTS recipients due to polyhydramnios. Mean (SD), two-tailed unpaired t test, ∗∗∗p ≤ 0.001. (D) Principal component analysis (PCA) of amniotic fluid metabolomics data using top 10% most variable metabolites. (E) Volcano plot showing differential metabolites in TTTS vs. control, including enrichment of fatty acids, arachidonic acid metabolites, and steroid hormones. Fold change threshold ≥2, FDR ≤0.001. See also Table S2 and Figure S3.

To evaluate fetal metabolic adaptations to cardiovascular stress, we collected midgestation amniotic fluid from TTTS (n = 22) and control (n = 10) pregnancies for untargeted metabolomic analysis (Figure 1A; Table S1). We selected severe TTTS pregnancies (stage III) with evidence of cardiovascular stress based on abnormal blood flow patterns in the umbilical artery, ductus venosus, or umbilical vein—vessels that are unique to the fetal circulation (Figure 1B).^19^ These vessels are routinely interrogated in monochorionic-diamniotic twins using Doppler ultrasound to detect when there is: 1) increased placental resistance and cardiac afterload demonstrated by absent or reversed end-diastolic flow in the umbilical artery and 2) right heart overload and failure manifested by absent or reversed flow in the ductus venosus a-wave or pulsatile flow in the umbilical vein.^20^ For TTTS cases, amniotic fluid was collected from the recipient twin sac at the time of fetoscopic laser surgery.^4^ Control samples were obtained from singleton pregnancies with normal genetic testing and no fetal anomalies detected on prenatal ultrasound, utilizing discarded genetic amniocentesis specimens.

Maternal age and gestational age were significantly different between groups (Table 1); however, these variables had no significant impact on metabolite profiles based on correlation analysis (Figure S1). Control patients tended to be older because the most common indication for genetic amniocentesis was advanced maternal age (35 years of age or greater). The earlier gestational age among controls is also not surprising given the timing of diagnostic genetic testing which often occurs earlier in gestation than laser surgery for TTTS. The distribution of fetal sex was similar in both groups. Of the TTTS pregnancies, 64% (14/22) of recipients and 50% (11/22) of donors had abnormal Doppler studies, indicating clinically evident cardiovascular stress in most of the TTTS twins. Detailed clinical characteristics are shown in Table S1.

**Table 1:** Clinical characteristics of the metabolomics cohort

Characteristic	Control (n = 10)	TTTS (n = 22)	*P*
Maternal age, years	33.7 (4.9)	26.7 (5.4)	0.002
Gestational age, weeks	18.1 (2.2)	19.6 (1.7)	0.049
Fetal sex, female	5 (50.0)	12 (54.5)	>0.99
Indication for amniocentesis^a^			
Advanced maternal age	2 (20)	–	
Abnormal screening test	5 (50)	–	
Other	4 (40)	–	
Disease stage			
Stage IIID	–	8 (36.4)	
Stage IIIR	–	11 (50)	
Stage IIIDR	–	3 (13.6)	

Data are mean (SD) or n (%).

Unpaired t test or χ^2^ test.

a Indications not mutually exclusive. See also Table S1.

### Excessive amniotic fluid in TTTS recipients dilutes amniotic fluid components

A critical clinical characteristic is aberrant amniotic fluid volume, given that a fluid imbalance is the first diagnostic sign of TTTS in monochorionic-diamniotic twin pregnancies. We used amniotic fluid from the recipient twin who has excess amniotic fluid (polyhydramnios) as a result of volume overload and increased fetal urine output. (Donor twin amniotic fluid is by definition low (oligohydramnios) and is not acquired during laser surgery.) To assess the extent to which volume affected the concentration of amniotic fluid metabolites, we measured amniotic fluid protein concentration (Figure 1C). The mean protein concentration was significantly lower in TTTS vs. control amniotic fluid (1.8 vs. 4.0 mg/mL, *p* = <0.001), suggesting a greater than 2-fold dilution of amniotic fluid components in TTTS, in agreement with our prior work.^21^^,^^22^ We therefore normalized metabolite data by protein concentration for each sample to adjust for dilution.

### Amniotic fluid metabolites reveal changes in fatty acid, glucose, and steroid hormone metabolism in TTTS

Untargeted liquid chromatography/mass spectrometry (LC/MS)-based profiling of amniotic fluid metabolites recognized >400 metabolites. TTTS cases grouped separately from controls based on principal component analysis (PCA), with controls clustered more closely together (Figure 1D). Among 133 differentially abundant metabolites (absolute fold change [FC] > 2, FDR ≤0.001), more than half exhibited low retention times upon chromatography employing an aqueous normal-phase matrix (e.g., fatty acids and lipids); a subset of these metabolites was effectively resolved and identified by targeted reverse-phase liquid chromatography (Table S2).

Capric acid, a saturated, medium-chain fatty acid, was the most abundant metabolite enriched in TTTS vs. control (log_2_FC 17.6, adjusted p = 8.32e^−20^; Figure 1E). Relative accumulation of several other saturated free fatty acids in TTTS amniotic fluid was also observed, including myristic and palmitic acids, which are known to promote adaptive cardiac growth and hypertrophy via AMP-activated protein kinase (AMPK) signaling.^23^^,^^24^^,^^25^ AICA (5-aminoimidazole-4-carboxamide) riboside (AICAR), an AMPK activator, had the second highest FC in TTTS vs. control amniotic fluid (log_2_FC 11.7, adjusted p = 3.89e^−6^). AICAR positively regulates glucose uptake in muscle^26^^,^^27^ and inhibits gluconeogenesis,^28^ as well as glycogen synthesis, in rodents.^29^ Notably, AICAR has been studied as a cardioprotective agent in animal models of cardiac ischemia.^30^^,^^31^^,^^32^

Amniotic fluid pyruvate was depleted in TTTS amniotic fluid, consistent with other changes in glucose metabolites. Also depleted was retinoic acid, an essential transcriptional regulator of genes involved in lipid and energy metabolism^33^^,^^34^ as well as the development of multiple organs, including the heart.^35^^,^^36^ Retinoic acid levels were previously shown to be reduced in human adult heart failure.^37^

Arachidonic acid and its derivatives (prostaglandins, thromboxane B2) were found to be enriched in TTTS. These polyunsaturated fatty acids (PUFAs; components of membrane phospholipids) are major mediators of inflammation, contribute to the regulation of vascular tone (reviewed in ref. ^38^^,^^39^), and have been associated with hypertension.^40^ Cell damage from oxygen-derived free radicals and lipid peroxidation might also affect levels of amniotic fluid PUFAs.

We also noted an increase in adrenal steroid hormones with glucocorticoid and mineralocorticoid activity, including aldosterone precursors, deoxycorticosterone, corticosterone, and cortexolone (also known as 11-deoxycortisol, the precursor of cortisol). Umbilical cord blood corticosterone has been associated with fetal stress in labor,^41^ and deoxycorticosterone is known to be produced in the fetal kidney.^42^

Other metabolites of interest enriched in TTTS amniotic fluid reflected potential effects on fetal organ systems other than the heart. Endocannabinoids, such as 2-arachidonoylglycerol and anandamide, are arachidonic acid-derived neuroregulators that bind CB1 cannabinoid receptors and play important roles in brain development, specifically in guidance of axon growth and synapse formation.^43^^,^^44^ Metabolites that accumulate in the setting of hepatobiliary injury or stasis, including bile acids (e.g., taurolithocholic acid) and pipecolinic acid (also known as pipecolic acid),^45^^,^^46^ were also increased in TTTS amniotic fluid.

Taken together, metabolic findings suggest that TTTS recipient twins are exposed to major shifts in metabolism in response to hemodynamic overload. These changes include fatty acid and glucose utilization, as well as activation of the fetal hypothalamic-pituitary-adrenal axis.

### Metabolite patterns identify distinct clusters of severe TTTS

**Figure 2:**
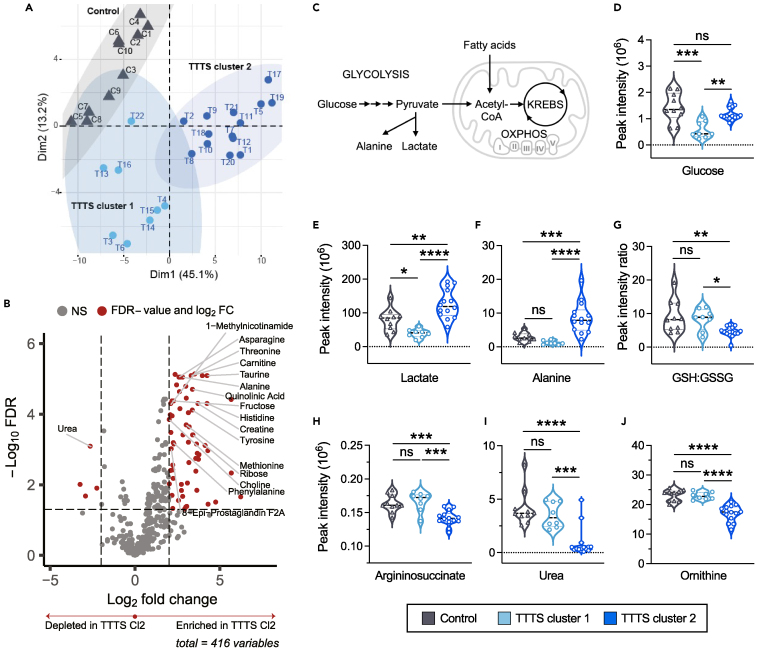
Distinct clusters of TTTS are defined by glycolytic metabolites, redox stress, and altered nitrogen metabolism (A) PCA of metabolite profiles using top 10% most variable metabolites within TTTS samples. (B) Volcano plot of differential metabolites between TTTS cluster 2 and TTTS cluster 1 showing accumulation of amino acids, carnitines, 8-*epi*-PGF_2α_, ATP and NAD+ metabolites. Fold-change threshold ≥2, FDR ≤0.001. See also Table S4. (C) Schematic overview of glycolysis and fatty acid oxidation pathways. (D–F) Normalized mass spectrometry peak intensities for glucose and glycolytic metabolites: glucose, lactate, and alanine. See also Figures S2A and S2B. (G) Reduced:oxidized glutathione (GSH:GSSG) suggest increased oxidative stress and redox imbalance in TTTS cluster 2 compared with TTTS cluster 1 and control. See also Figures S2C–S2G. (H–J) Normalized mass spectrometry peak intensities for urea cycle intermediates: argininosuccinate, urea, and ornithine. See also Figures S2H–S2J. One-way ANOVA with Tukey’s multiple comparisons test, ∗p ≤ 0.05, ∗∗p ≤ 0.01, ∗∗∗p ≤ 0.001, ∗∗∗∗p ≤ 0.0001.

Compared to the control group, amniotic fluid metabolite profiles were highly variable within the TTTS group, suggesting significant heterogeneity within the disease group. To further investigate this heterogeneity, we reclustered the samples using the most variable metabolites in TTTS amniotic fluid (top 10%). Two subgroups emerged, which we labeled as TTTS cluster 1 (n = 8) and TTTS cluster 2 (n = 14; Figure 2A). The clusters were not clearly distinguishable by clinical characteristics (Table 2; case details are provided in Table S3). Maternal age, maternal body mass index, gestational age, fetal sex, and ultrasound parameters were similar between clusters, including the frequency of abnormal Doppler studies that are reflective of cardiac strain, although three recipients from cluster 2 had evidence of mitral or tricuspid regurgitation compared with none from cluster 1. Of note, none of the pregnancies was complicated by maternal pregestational or gestational diabetes or hypertension (potential confounders). One pregnancy from each cluster was complicated by twin anemia-polycythemia sequence (TAPS).^47^

**Table 2:** Clinical characteristics of TTTS clusters

Characteristic	Cluster 1 (n = 8)	Cluster 2 (n = 14)	*P*
Maternal age, y	26.9 (7.4)	26.6 (4.1)	0.93
Maternal BMI, kg/m^2^, median (IQR)	26.5 (25.7–29.5)	24.9 (21.4–35.1)	0.57
Gestational age at laser, weeks	19.6 (1.8)	19.6 (1.7)	0.97
Fetal sex, female	5 (62.5)	7 (50.0)	0.67
Disease stage	–	–	0.45
Stage IIID	3 (37.5)	5 (37.5)	
Stage IIIR	3 (37.5)	8 (57.1)	
Stage IIIDR	2 (25.0)	1 (7.1)	
Recipient MVP, cm, median (IQR)	9.4 (8.4–12.7)	11.5 (9.4–13.9)	0.34
Recipient mitral or tricuspid regurgitation	0	3 (21.4)	0.27
Recipient Doppler abnormality	5 (62.5)	9 (64.3)	>0.99
Absent end-diastolic flow in the UA	1 (12.5)	4 (28.6)	
Absent/reversed flow in the DV a-wave	4 (50.0)	8 (57.1)	
Pulsatile flow in the UV	3 (37.5)	8 (57.1)	
Donor Doppler abnormality	5 (62.5)	6 (42.9)	0.66
Absent end-diastolic flow in the UA	5 (62.5)	8 (57.1)	
Absent/reversed flow in the DV a-wave	2 (25.0)	2 (14.3)	
Pulsatile flow in the UV	1 (12.5)	3 (21.4)	
Discordance, mean % difference	25.9 (11.2)	22.9 (15.4)	0.40
Twin anemia-polycythemia sequence	1 (12.5)	1 (7.1)	>0.99

D, donor; DV, ductus venosus; MVP, maximum vertical pocket; R, recipient; UA, umbilical artery; UV umbilical vein. See also Table S3.

Data are n (%) or mean (SD) unless otherwise specified.

Unpaired t test, Fisher’s exact test, or Mann-Whitney test.

Differential metabolite analyses revealed the enrichment of several amino acids in TTTS cluster 2 compared with cluster 1, including alanine, aspartate, methionine, and tyrosine (Figure 2B; Table S4). Carnitine and its derivatives, important cofactors for the metabolism of long-chain fatty acids, were also increased in cluster 2, suggesting potential pressure to increase the transport capacity of fatty acids into mitochondria for β-oxidation. A change in energy metabolism in cluster 2 was also supported by the enrichment of ATP precursors or building blocks (creatine and ribose) and metabolites involved in the production of NAD+ (1-methylnicotinamide, quinolinic acid), an essential substrate for redox reactions.^48^ Accumulation of the isoprostane 8-*epi*-prostaglandin F2α (8-*epi*-PGF_2α_) in cluster 2 was also notable as 8-*epi*-PGF_2α_ is a product of lipid peroxidation and arachidonic acid metabolism and an *in vivo* biomarker of oxidative stress^49^^,^^50^ that increases in response to angiotensin II- and norepinephrine-induced hypertension.^51^

### TTTS clusters are defined by glycolytic metabolites, redox stress, and altered nitrogen metabolism

To construct a coherent model of the metabolic changes observed in TTTS recipients, we next considered the metabolic remodeling that occurs in the failing postnatal heart. With increased oxygen availability after birth, the heart switches from using glucose and lactate as the primary energy substrates in fetal life to fatty acid oxidation for mitochondrial ATP production postnatally. Under conditions of stress, the postnatal heart reverts to fetal-like metabolic programs,^52^^,^^53^ switching back to the primary utilization of glucose as fuel. This metabolic flexibility endows the postnatal heart with the ability to compensate and restore energy homeostasis in order to maintain function. The fetal heart relies on glucose at baseline, and therefore, cannot activate the same “switch” in metabolism as the postnatal heart. The degree to which the baseline preference of the fetal heart for glucose affects metabolic flexibility and cardiac adaptation to stress is not known.

To evaluate the effects of cardiac stress on glycolysis, we first examined glycolytic metabolites (Figures 2C–2F, S2A, and S2B) by cluster. Compared with controls, normalized amniotic fluid glucose levels were lower in TTTS clusters 1 and 2 (Figure 2D), while pyruvate and acetyl- coenzyme A (CoA) levels were similar between all groups (Figures S2A and S2B). Lactate and alanine, the other products of anaerobic glycolysis,^54^ were increased in TTTS cluster 2 compared with cluster 1 and control (Figures 2E and 2F). Although stable isotope flux studies would be needed for definitive information regarding differential glucose metabolism, these results suggest a selective acceleration of glycolysis in TTTS cluster 2 cases.

To assess the redox state associated with these apparent shifts in energy substrate metabolism, we evaluated relative levels of glutathione in its reduced (GSH) and oxidized forms (GSSG). The GSH:GSSG ratio was significantly reduced in TTTS cluster 2 compared with cluster 1 and control amniotic fluid (Figure 2G), indicating a more profound redox imbalance in cluster 2. Notably, these differences appeared to be driven by depletion of GSH (Figures S2C and S2D). Cluster 2 also exhibited an increased lactate:pyruvate ratio (Figure S2E). Elevations in the extracellular lactate:pyruvate ratio are correlated with reduced tissue oxygen tension^55^^,^^56^ and reflective of the intracellular NADH:NAD+ ratio,^57^^,^^58^ an indicator of redox potential. Accumulation of antioxidants, such as taurine and ergocalciferol (vitamin D2), was also observed in cluster 2 (Figures S2F and S2G), potentially reflecting compensatory mechanisms. These data, along with the accumulation of amniotic fluid 8-*epi*-PGF_2α_, are consistent with enhanced oxidative stress in TTTS cluster 2 vs. cluster 1.

Disruptions in cellular homeostasis and energy metabolism are tightly linked to disruptions in protein synthesis and degradation.^59^ We therefore investigated amino acid and nitrogen metabolism by considering relative levels of urea cycle intermediates (Figures 2H–2J and S2H–S2J). The urea cycle ultimately converts ammonia generated by amino acid metabolism to urea (Figure S2H). Compared to controls and cluster 1, TTTS cluster 2 exhibited a significant depletion of argininosuccinate, urea, and ornithine (Figures 2H–2J), while no difference was observed in arginine levels (Figure S2I). Of the urea cycle intermediates, citrulline was uniquely increased in cluster 2 (Figure S2J), suggesting impaired urea cycle flux. Alternatively, citrulline is produced during the conversion of arginine to nitric oxide (Figure S2H); thus elevated citrulline levels might reflect an increased generation of nitric oxide, a pivotal mediator of vasodilation in the setting of fetal hypertension.

Collectively, the fluid metabolite pattern associated with TTTS cluster 2 suggests increased glycolytic activity in the setting of redox stress and reduced amino acid utilization and metabolism compared with cluster 1.

### Perinatal outcomes associated with TTTS metabolic clusters

**Figure 3:**
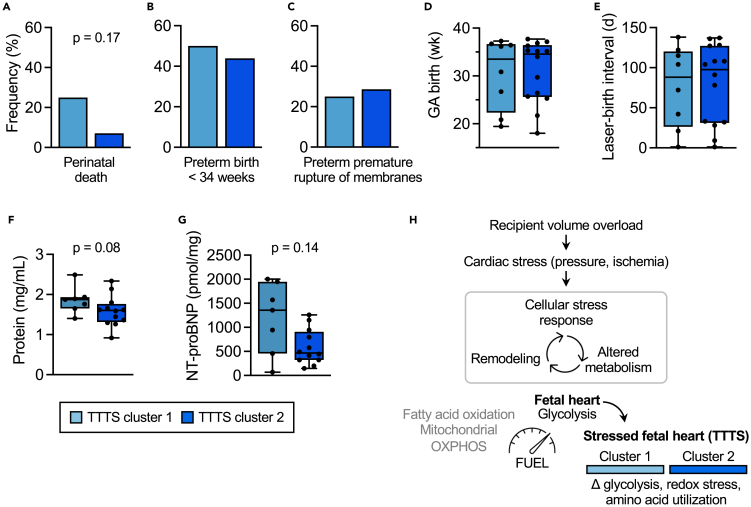
Perinatal outcomes by TTTS cluster (A–C) Similar frequency of perinatal death, early preterm birth (prior to 34 weeks of gestation), and preterm premature rupture of membranes between clusters. See also Table S5. (D and E) Similar median gestational age at birth and interval between laser surgery and birth (latency in days). See also Table S5. (F) Amniotic fluid protein levels stratified by cluster (data also shown in Figure 1C). (G) Normalized amniotic fluid NT-proBNP concentration determined by ELISA. (H) Model of fetal metabolic adaptation to cardiac stress. Data are % or median (IQR). Fisher’s exact test or Mann-Whitney test.

We sought to correlate the metabolomics results with clinical outcomes for TTTS clusters 1 and 2. Frequencies of perinatal death, early preterm birth (<34 weeks of gestation), and preterm premature rupture of membranes were similar between cluster 1 and 2 (Figures 3A–3C; Table S5). Median gestational age at birth and the time interval between laser therapy and birth were also similar (Figures 3D and 3E; Table S5). We further compared amniotic fluid protein and N-terminal pro-brain natriuretic peptide (NT-proBNP; released by cardiomyocytes in response to stretch^60^) between clusters to assess potential subclinical differences in volume overload; these were similar between groups (Figures 3F and 3G). An important consideration is that all TTTS cases were treated with fetoscopic laser ablation of placental vessels to effectively divide the fetal circulations and treat the underlying cause of the disease. Therefore, similarities in clinical outcomes might be attributed to the success of laser therapy rather than a lack of biologically relevant difference between clusters. Collectively, our data demonstrate significant metabolic remodeling in the setting of recipient volume overload and cardiovascular stress in TTTS (Figure 3H) with still uncertain implications for future childhood health.

## Discussion

We leveraged severe TTTS as a model to explore potential metabolic changes in fetuses under cardiovascular stress from volume overload. Metabolomic analyses of amniotic fluid revealed accumulation of free fatty acids, adrenal stress hormones, biomarkers of oxidative stress, and concomitant reduction in pyruvate, suggesting significant changes in energy metabolism in TTTS. Using PCA of the top differential metabolites, we uncovered two distinct clusters of TTTS (cluster 1 and cluster 2). These clusters were characterized by differences in markers of glycolysis, redox state, and amino acid metabolism.

TTTS cluster 2 showed more pronounced differences in amniotic fluid metabolites than TTTS cluster 1 when compared with control. The suggested reliance on glycolysis in TTTS cluster 2 is a hallmark of metabolic remodeling of the stressed postnatal heart.^61^ Our data also suggest that fetuses, which rely on glycolysis at baseline, are able to further augment glycolytic flux in response to cardiovascular stress. This adaptive response was associated with an imbalance in the redox state and evidence of activation of compensatory mechanisms to restore homeostasis based on accumulation of NAD+ metabolites and antioxidants in the amniotic fluid. We speculate that multiple factors may contribute to the notable accumulation of amino acids and reduction in urea in cluster 2, including differences in the fetal response to: 1) hepatocyte injury and mitochondrial dysfunction due to right heart failure leading to urea cycle defects and 2) cellular stress response mechanisms that alter amino acid metabolism and reduce overall protein synthesis.^62^^,^^63^ Although significant differences in signs of cardiac failure (NT-proBNP, mitral or tricuspid regurgitation) were not found, our small sample size limited our ability to detect differences in certain variables.

One insight gleaned from this study is the variability in fetal metabolic adaptation to cardiovascular stress. Whether this heterogeneity in TTTS represents fetuses at different points along the same spectrum (i.e., early vs. late or milder vs. more severe disease physiology) or innate differences in the ability of individual fetuses to adapt is uncertain. Although changes in amniotic fluid metabolites for cluster 2 were more prominent than cluster 1, cluster 2 changes are not necessarily indicative of worse disease. On the contrary, cluster 2 fetuses may be exhibiting greater flexibility and compensation than cluster 1 fetuses. In other words, cluster 2 may represent an adaptive response, whereas cluster 1 may be maladaptive. Nevertheless, we propose that recognition of disease subtypes is an important first step toward understanding variations in clinical presentation and factors associated with perinatal and longer-term adverse health outcomes. Although we did not find differences in perinatal outcomes between the two TTTS clusters, we propose that differences in intermediary metabolism might have more subtle clinical significance. For instance, the adequacy of the fetal adaptive response may be related to the gestational age at diagnosis and the unpredictable rate of disease progression. Long-term impacts on cardiovascular conditions cannot be ruled out either, due to our current limited collection of TTTS samples.

Our work integrates and builds upon previous studies that have examined metabolic remodeling in postnatal heart failure and molecular alterations in TTTS amniotic fluid. A recent study identified mitochondrial dysfunction, endoplasmic reticulum stress, and oxidative stress as defining features of early heart failure in patients and mice with hypoplastic left heart syndrome,^64^ suggesting similarities between fetal and postnatal metabolic features associated with cardiovascular stress. Relatively few studies have reported results from human amniotic fluid metabolomics, and those studies have focused on preterm birth.^65^^,^^66^^,^^67^^,^^68^^,^^69^ One study that used LC/MS-based metabolomics to assess TTTS recipient amniotic fluid investigated differences in metabolite profiles according to cardiac function and pre-vs. post-laser sampling.^70^ Notably, prior to laser treatment, metabolites that negatively correlated with cardiac function (myocardial performance index) included acyl carnitines, fatty acids, ceramides, lipids, and hormones. Our data showing accumulation of similar metabolites in TTTS vs. control validate these observations. We did not, however, observe significant differences in these metabolites between TTTS clusters, perhaps reflecting the clinical similarity between samples in our cohort. Our use of an unsupervised bioinformatic approach to cluster cases revealed that stratification using clinical variables may not adequately reflect the underlying biology.

We and others have demonstrated increases in heart failure biomarkers^21^^,^^71^ and steroid hormone levels^22^ in TTTS recipient amniotic fluid compared with controls. We have also previously observed elongation of the umbilical artery in recipient twins associated with cardiac dysfunction, suggesting potential fetal vascular remodeling.^21^ These data, together with the results from the present study, raise the question of the long-term impacts of *in utero* stress on TTTS survivors. This question requires further investigation.

### Limitations of the study

In spite of the exciting new observations, there are several important caveats to consider. First, TTTS twins have a shared circulatory system. Therefore, donor twin physiology, such as upregulation of RAAS factors due to hypovolemia,^72^^,^^73^ may affect recipient twin biology in a manner specific to TTTS fetuses. Secondly, we have interpreted our results through the lens of the recipient’s cardiovascular biology which includes volume overload, hypertrophy, and heart failure. In this regard, we acknowledge that the recipient fluid has inputs from many sources, including organ systems other than the heart, such as the placental membranes and the maternal circulation (Figure S3). We are also aware that amniotic fluid analysis provides only a snapshot of a complex and dynamic system of intracellular metabolic fluxes reflected in the extracellular environment. Complete amniotic fluid turnover occurs every 48 h;^74^ thus, our “snapshots” represent metabolites that have recently accumulated. Third, we recognize the limitations of the sample size, the use of unmatched singleton controls (amniotic fluid from healthy twin pregnancies was unavailable for investigation), and the potential for confounding. Lastly, because every TTTS case was treated with laser ablation to separate the circulations of the donor and the recipient, the use of amniotic fluid metabolite signatures to predict perinatal outcomes is limited. In the future, a longitudinal cohort needs to be enrolled to collect amniotic fluid samples from two time points, pre-laser and at birth, to assess whether metabolic differences persist after definitive laser therapy.

In summary, our study presents an attempt to assess metabolic reprogramming in the human fetus experiencing cardiovascular stress. Notwithstanding some limitations, amniotic fluid provides an invaluable opportunity to assess molecular aspects of human fetal development and disease. This applies especially to diseases for which animal models are not available. Advancing knowledge of fetal plasticity and adaptability is critical for understanding how *in utero* insults impact future health. Clarifying the thresholds at which fetal adaptive responses become maladaptive and injurious is equally important. We suspect innate differences in fetal adaptability exist given the wide-ranging clinical presentations observed. Lastly, a more comprehensive picture of the molecular pathophysiology of TTTS is the first step toward changing our current inability to predict disease progression and complications after laser therapy. Future research efforts correlating fetal molecular studies with longer-term patient outcomes are needed to determine the extent to which fetal programming and the DOHaD hypothesis apply to TTTS and other fetal conditions.

## STAR★Methods

### Key resources table

**Table:** 

REAGENT or RESOURCE	SOURCE	IDENTIFIER
**Biological samples**		

Human amniotic fluid	UTHealth Houston Fetal Center Biobank	N/A

**Chemicals, peptides, and recombinant proteins**		

Acetonitrile Optima LC/MS, 4L	Fisher Chemical	Cat#A955-4
Acetic Acid, Glacial, OmniTrace Ultra, 250mL	EMD Millipore	Cat#AX0078/7
Ammonium acetate,99.999% trace metal basis	Sigma-Aldrich	Cat#372331-10mg
Methanol Optima LC/MS, 4L	Fisher Chemical	Cat#A456-4
2-PropanolOptima LC/MS, 2.5L	Fisher Chemical	Cat#A461-212
Ethylenediaminetetraacetic acid 99.995% trace metals basis	Sigma-Aldrich	Cat#431788-25g
Methyl tert-butyl ether	Fischer Scientific	Cat# 60-016-44
Ammonium formate	Sigma-Aldrich	Cat# 70221-25G-F
Formic acid	MilliPore Sigma	Cat# 5330020050
NaOH	VWR	Cat# EM1.06498.1000

**Critical commercial assays**		

DC protein assay	Bio-Rad	Cat#5000116
Pierce BCA protein assay	ThermoFisher Scientific	Cat#23225
Alpco NT-Pro BNP ELISA	Fisher Scientific	Cat#04BI20852W

**Deposited data**		

Raw and analyzed data	This paper	Raw metabolomics data is available at the NIH Common Fund's National Metabolomics Data Repository (NMDR) website, the Metabolomics Workbench^77^: https://doi.org/10.21228/M8513X (Study ID ST002797). See Tables S2 and S4.

**Software and algorithms**		

MassHunter Qualitative Analysis (B10.0 spk1)	Agilent Technology, Santa Clara, CA	N/A
MassProfinder 8.0	Agilent Technology, Santa Clara, CA	N/A
MassProfilerProfessional (v15.1)	Agilent Technology, Santa Clara, CA	N/A
GraphPad Prism version 9.4.1	GraphPad	https://www.graphpad.com/
R code for bioinformatics analysis	GitHub	https://github.com/FanBioinformatics/TTTS_iScience_metabolome.git
R version 4.2.1 or higher	The R Project for Statistical Computing	https://www.r-project.org/
limma v3.54.2	Matthew E. Ritchie et al. (2015)^75^	https://doi.org/10.1093/nar/gkv007
factoextra v1.0.7	CRAN – R package	http://www.sthda.com/english/rpkgs/factoextra

### Resource availability

#### Lead contact

Further information and requests for resources should be directed to and will be fulfilled by the lead contact, Ramesha Papanna (ramesha.papanna@uth.tmc.edu).

#### Materials availability

This study did not generate new unique reagents.

### Experimental model and study participant details

#### Human participants

We analyzed banked and discarded human amniotic fluid samples for this study. Deidentified participant data are provided in Tables S1 and S3. This study was approved by the Institutional Review Board of the McGovern Medical School at The University of Texas Health Science Center at Houston (#HSC-MS-13-0712).

### Method details

#### Study design

We set out to perform an exploratory study that compared second-trimester amniotic fluid from monochorionic-diamniotic twin pregnancies complicated by stage III TTTS and singleton controls. The study cohort comprised a convenience sample of cryopreserved specimens. Included TTTS cases were selected from a well-characterized subset with: 1) sonographic findings consistent with stage III disease: polyhydramnios in the recipient sac (maximum vertical pocket of fluid >8 cm); oligohydramnios in the donor sac (maximum vertical pocket <2 cm); bladder not visible in the donor; and abnormal Doppler evaluation in either twin (absent or reversed end-diastolic flow in the umbilical artery, reversed ductus venosus a-wave flow, or pulsatile umbilical vein flow),^19^ and 2) complete clinical and outcomes data. Controls with normal genetic testing (karyotype or chromosomal microarray) and normal anatomical ultrasound were included. All TTTS patients underwent comprehensive ultrasound examination with Doppler studies prior to surgery for diagnosis and staging per protocol; however, genetic testing results were not available for all cases (testing is not routinely performed for non-anomalous twins with TTTS). Pregnancies with apparent genetic or structural abnormalities were excluded.

#### Amniotic fluid and clinical data collection

Amniotic fluid was previously collected and banked from pregnant individuals with monochorionic-diamniotic twins complicated by TTTS undergoing fetoscopic laser ablation of placental anastomoses at the UTHealth Houston Fetal Center at Children’s Memorial Hermann Hospital. Samples were collected from the recipient twin sac immediately upon entry with the operative cannula and prior to placental laser ablation or amnioinfusion for improving the visualization. The amniotic fluid was centrifuged, and the supernatant was stored at −80°C for future use. Frozen genetic amniocentesis samples, discarded from further analyses, served as controls. For TTTS cases, demographics, clinical characteristics, and outcomes were abstracted from the Fetal Center research database which is maintained by trained research staff. For controls, available clinical variables were limited to maternal age, gestational age, indication for amniocentesis, and genetic testing results.

#### Untargeted metabolite profiling

##### Metabolite extraction

Amniotic fluid metabolites were extracted by addition of 1 part amniotic fluid to 15 parts 70% acetonitrile in ddH2O (vol:vol). The mixture was briefly vortexed and then centrifuged for 5 min at 16,000 × *g* to pellet precipitated proteins. The protein pellet was solubilized in 0.2M NaOH and quantified by DC Protein Assay (Bio-Rad). The volume of metabolite extract was normalized by amniotic protein content. An aliquot of the resulting extract (3 μL) was subjected to LC/MS untargeted metabolite profiling in positive and negative ion modes as described previously described,^76^ using a platform comprised of an Agilent Model 1290 Infinity II liquid chromatography system coupled to an Agilent 6550 iFunnel time-of-flight MS analyzer.

##### Aqueous normal phase LC/MS

Chromatography of metabolites utilized aqueous normal phase (ANP) chromatography on a Diamond Hydride column (Microsolv). Mobile phases consisted of: (A) 50% isopropanol, containing 0.025% acetic acid, and (B) 90% acetonitrile containing 5 mM ammonium acetate. To eliminate the interference of metal ions on chromatographic peak integrity and electrospray ionization, EDTA was added to the mobile phase at a final concentration of 6 μM. The following gradient was applied: 0-1.0 min, 99% B; 1.0-15.0 min, to 20% B; 15.0 to 29.0, 0% B; 29.1 to 37min, 99% B.

##### Reversed-phase LC/MS

We performed reversed-phase LC/MS to confirm targeted hydrophobic metabolites that eluted early in the ANP column and showed a significant difference between groups. Extraction of lipids and fatty acids was carried out using cold methanol, methyl tert-butyl ether (MTBE), and water. Specifically, 10 μL amnionic fluid was added to 225 μL cold methanol, vortexed for 10 seconds, then 750 μL of cold MTBE was added. The mixture was vortexed for 10 seconds and shaken for 6 minutes at 4°C. Phase separation was induced by adding 188 μL of LC/MS-grade water followed by centrifugation at 14,000 rpm for two minutes. The lipid phase was collected and dried under vacuum. Dried lipid extracts were normalized to protein content and resuspended in methanol/toluene (9:1, v/v) mixture for positive and negative detection by LC/MS.

Lipid extracts were also analyzed by LC/MS, using a platform comprised of an Agilent Model 1290 Infinity II liquid chromatography system coupled to an Agilent 6550 iFunnel time-of-flight MS analyzer. Lipids were separated by reversed phase chromatography on a ZORBAX EclipsePlus C18 (100 x 2.1 mm, 1.8 μm, Agilent Technologies). For positive ion mode, mobile phases consisted of: (A) 60:40 (v/v) acetonitrile: H2O containing 10 mM ammonium formate and 0.1% formic acid, and (B) 90:10 (v/v) isopropanol:acetonitrile containing 10mM ammonium formate and 0.1% formic acid. Column temperature was 60°C and sample injection volume was 2 μL. The following gradient was applied: 0 min, 15% B; 0-2.0 min, to 30% B; 2.0 to 2.5 min, to 48% B; 2.5-8.5 min, to 72% B; 8.5 to 11.5 min, to 99% B; 11.5 to 12 min, 99% B; 12.1 to 15 min, 15% B. The flow rate was 0.6 mL/min. LC conditions for negative ion mode are the same as positive mode, except that 10 mM ammonium acetate was replaced by 10 mM ammonium acetate.

The following mass spectrometer parameters were used for both positive and negative ion modes: drying gas temperature was set at 200°C with a flow rate of 14 L/min. Nebulizer pressure was at 35 psi. Sheath gas temperature was set at 350°C with a flow rate of 11 L/min. Capillary and nozzle voltage were at 3500v and 1000v, respectively. Mass spectra were acquired at 2 spectra/sec over the range of 100 to 1700 m/z.

##### Metabolite identification

Raw LC/MS data were analyzed using MassHunter Profinder 8.0 and MassProfiler Professional (MPP) 15.1 software (Agilent Technologies). To ascertain the identities of metabolites, LC/MS data were searched against an in-house annotated personal metabolite database created using MassHunter PCDL manager 8.0 (Agilent) based on monoisotopic neutral mass (<5 ppm mass accuracy) and chromatographic retention times of pure standards. A molecular formula generator (MFG) algorithm in MPP was used to generate and score empirical molecular formulae, based on a weighted consideration of monoisotopic mass accuracy, isotope abundance ratios, and spacing between isotope peaks. A tentative compound ID was assigned when the PCDL database and MFG scores concurred for a given candidate molecule. Tentatively assigned molecules were confirmed based on a match of LC retention times and/or MS/MS fragmentation spectra for pure molecular standards.

#### Amniotic fluid biochemical analysis

Amniotic fluid protein concentration was determined using the Pierce BCA protein assay (Thermo Fisher Scientific). Amniotic fluid NT-proBNP concentration was measured using the Alpco NT-Pro BNP ELISA (Fisher Scientific 04BI20852W) and normalized to amniotic fluid protein levels.

### Quantification and statistical analysis

#### Metabolomics data analysis

Normalized concentrations for metabolites were first log_2_-transformed. R package limma^75^ was then applied to fit a linear regression model to identify differential metabolites using empirical Bayes statistics. Differential metabolites were thus calculated based on a log_2_ fold-change threshold of ≥ 2 and FDR of ≤ 0.001. Principal component analysis (PCA) was carried out using R package factoextra based on the top 50 most variable metabolites across all the samples as well as across all the diseased samples.

#### Clinical data analysis

Clinical data are presented as n (%), mean (SD), or median (IQR) as specified in the figure and table legends. Fisher’s exact test or χ^2^ test were used for categorical variables. Two-tailed unpaired t test, Mann-Whitney test, or one-way ANOVA with Tukey’s multiple comparisons test were used for continuous variables as appropriate. Statistical significance was defined as *p* <0.05 unless otherwise specified. Statistical details are specified in the figure and table legends. GraphPad Prism software (version 9.4.1) and R (version 4.2.1 or higher) were used for analyses.

## Data Availability

• Metabolomics data have been deposited at the NIH Common Fund's National Metabolomics Data Repository (NMDR) website, the Metabolomics Workbench and are publicly available. Study ID and DOI are listed in the key resources table. See also Tables S2 and S4. • All original code has been deposited at GitHub and is publicly available. DOIs are listed in the key resources table. • Any additional information required to reanalyze the data reported in this paper is available from the lead contact upon request.

## Supplemental information

Document S1. Figures S1–S3 and Tables S1, S3, and S5

Table S2. Differentially abundant metabolites - TTTS vs. Control, related to Figure 1

Table S4. Differentially abundant metabolites - TTTS Cluster 2 vs. TTTS Cluster 1, related to Figures 2 and S2

## References

[1] Mahieu-Caputo D., Dommergues M., Delezoide A.L., Lacoste M., Cai Y., Narcy F., Jolly D., Gonzales M., Dumez Y., Gubler M.C. (2000). Twin-to-twin transfusion syndrome. Role of the fetal renin-angiotensin system. Am. J. Pathol..

[2] Bajoria R., Ward S., Sooranna S.R. (2004). Influence of vasopressin in the pathogenesis of oligohydramnios-polyhydramnios in monochorionic twins. Eur. J. Obstet. Gynecol. Reprod. Biol..

[3] Senat M.-V., Deprest J., Boulvain M., Paupe A., Winer N., Ville Y. (2004). Endoscopic laser surgery versus serial amnioreduction for severe twin-to-twin transfusion syndrome. N. Engl. J. Med..

[4] Simpson L.L., Society for Maternal-Fetal Medicine (2013). Twin-twin transfusion syndrome. Am. J. Obstet. Gynecol..

[5] Wohlmuth C., Agarwal A., Stevens B., Johnson A., Moise K.J., Papanna R., Donepudi R., Bell C.S., Averiss I.E., Gardiner H.M. (2020). Fetal ventricular strain in uncomplicated and selective growth-restricted monochorionic diamniotic twin pregnancies and cardiovascular response in pre-twin-twin transfusion syndrome. Ultrasound Obstet. Gynecol..

[6] Pedra S.R.F.F., Smallhorn J.F., Ryan G., Chitayat D., Taylor G.P., Khan R., Abdolell M., Hornberger L.K. (2002). Fetal cardiomyopathies: pathogenic mechanisms, hemodynamic findings, and clinical outcome. Circulation.

[7] Tarca A.L., Romero R., Pique-Regi R., Pacora P., Done B., Kacerovsky M., Bhatti G., Jaiman S., Hassan S.S., Hsu C.-D., Gomez-Lopez N. (2020). Amniotic fluid cell-free transcriptome: a glimpse into fetal development and placental cellular dynamics during normal pregnancy. BMC Med. Genom..

[8] Hui L., Wick H.C., Moise K.J., Johnson A., Luks F., Haeri S., Johnson K.L., Bianchi D.W. (2013). Global gene expression analysis of amniotic fluid cell-free RNA from recipient twins with twin-twin transfusion syndrome. Prenat. Diagn..

[9] Schuchardt E.L., Miyamoto S.D., Crombleholme T., Karimpour-Fard A., Korst A., Neltner B., Howley L.W., Cuneo B., Sucharov C.C. (2022). Amniotic Fluid microRNA in Severe Twin-Twin Transfusion Syndrome Cardiomyopathy-Identification of Differences and Predicting Demise. J. Cardiovasc. Dev. Dis..

[10] Willner E.C., Galan H.L., Cuneo B.F., Hoffman H.A., Neltner B., Schuchardt E.L., Karimpour-Fard A., Miyamoto S.D., Sucharov C.C. (2021). Amniotic fluid microRNA profiles in twin-twin transfusion syndrome with and without severe recipient cardiomyopathy. Am. J. Obstet. Gynecol..

[11] Zwemer L.M., Bianchi D.W. (2015). The amniotic fluid transcriptome as a guide to understanding fetal disease. Cold Spring Harb. Perspect. Med..

[12] Barker D.J.P., Thornburg K.L. (2013). The obstetric origins of health for a lifetime. Clin. Obstet. Gynecol..

[13] Godfrey K.M., Gluckman P.D., Hanson M.A. (2010). Developmental origins of metabolic disease: life course and intergenerational perspectives. Trends Endocrinol. Metab..

[14] Rodríguez-Rodríguez P., Ramiro-Cortijo D., Reyes-Hernández C.G., López de Pablo A.L., González M.C., Arribas S.M. (2018). Implication of oxidative stress in fetal programming of cardiovascular disease. Front. Physiol..

[15] Sadek H., Olson E.N. (2020). Toward the goal of human heart regeneration. Cell Stem Cell.

[16] Van Mieghem T., Klaritsch P., Doné E., Gucciardo L., Lewi P., Verhaeghe J., Lewi L., Deprest J. (2009). Assessment of fetal cardiac function before and after therapy for twin-to-twin transfusion syndrome. Am. J. Obstet. Gynecol..

[17] Harbison A.L., Pruetz J.D., Ma S., Sklansky M.S., Chmait R.H., DeVore G.R. (2021). Evaluation of cardiac function in the recipient twin in successfully treated twin-to-twin transfusion syndrome using a novel fetal speckle-tracking analysis. Prenat. Diagn..

[18] Razeghi P., Young M.E., Alcorn J.L., Moravec C.S., Frazier O.H., Taegtmeyer H. (2001). Metabolic gene expression in fetal and failing human heart. Circulation.

[19] Quintero R.A., Morales W.J., Allen M.H., Bornick P.W., Johnson P.K., Kruger M. (1999). Staging of twin-twin transfusion syndrome. J. Perinatol..

[20] Baschat A.A. (2011). Examination of the fetal cardiovascular system. Semin. Fetal Neonatal Med..

[21] Donepudi R., Mann L.K., Wohlmuth C., Johnson A., Bebbington M.W., Moise K.J., Boudreaux D.S., Gardiner H., Papanna R. (2017). Recipient umbilical artery elongation (redundancy) in twin-twin transfusion syndrome. Am. J. Obstet. Gynecol..

[22] Hoffman M., Mann L.K., Won J.H., Bergh E.P., Donepudi R., Johnson A., Moise K.J., Macpherson C., Thom E., Mesiano S., Papanna R. (2020). Steroid Hormone Levels in Recipient Amniotic Fluid in Twin-Twin Transfusion Syndrome and Their Association with Preterm Delivery. Am. J. Perinatol..

[23] Riquelme C.A., Magida J.A., Harrison B.C., Wall C.E., Marr T.G., Secor S.M., Leinwand L.A. (2011). Fatty acids identified in the Burmese python promote beneficial cardiac growth. Science.

[24] Sundström J., Lind L., Vessby B., Andrén B., Aro A., Lithell H. (2001). Dyslipidemia and an unfavorable fatty acid profile predict left ventricular hypertrophy 20 years later. Circulation.

[25] Adrian L., Lenski M., Tödter K., Heeren J., Böhm M., Laufs U. (2017). AMPK Prevents Palmitic Acid-Induced Apoptosis and Lipid Accumulation in Cardiomyocytes. Lipids.

[26] Merrill G.F., Kurth E.J., Hardie D.G., Winder W.W. (1997). AICA riboside increases AMP-activated protein kinase, fatty acid oxidation, and glucose uptake in rat muscle. Am. J. Physiol..

[27] Iglesias M.A., Ye J.-M., Frangioudakis G., Saha A.K., Tomas E., Ruderman N.B., Cooney G.J., Kraegen E.W. (2002). AICAR administration causes an apparent enhancement of muscle and liver insulin action in insulin-resistant high-fat-fed rats. Diabetes.

[28] Vincent M.F., Marangos P.J., Gruber H.E., Van den Berghe G. (1991). Inhibition by AICA riboside of gluconeogenesis in isolated rat hepatocytes. Diabetes.

[29] Wojtaszewski J.F.P., Jørgensen S.B., Hellsten Y., Hardie D.G., Richter E.A. (2002). Glycogen-dependent effects of 5-aminoimidazole-4-carboxamide (AICA)-riboside on AMP-activated protein kinase and glycogen synthase activities in rat skeletal muscle. Diabetes.

[30] Kingma J.G., Simard D., Rouleau J.R. (1994). Timely administration of AICA riboside reduces reperfusion injury in rabbits. Cardiovasc. Res..

[31] Bolling S.F., Groh M.A., Mattson A.M., Grinage R.A., Gallagher K.P. (1992). Acadesine (AICA-riboside) improves postischemic cardiac recovery. Ann. Thorac. Surg..

[32] Galiñanes M., Bullough D., Mullane K.M., Hearse D.J. (1992). Sustained protection by acadesine against ischemia- and reperfusion-induced injury. Studies in the transplanted rat heart. Circulation.

[33] Li Y., Wong K., Walsh K., Gao B., Zang M. (2013). Retinoic acid receptor β stimulates hepatic induction of fibroblast growth factor 21 to promote fatty acid oxidation and control whole-body energy homeostasis in mice. J. Biol. Chem..

[34] Klyuyeva A.V., Belyaeva O.V., Goggans K.R., Krezel W., Popov K.M., Kedishvili N.Y. (2021). Changes in retinoid metabolism and signaling associated with metabolic remodeling during fasting and in type I diabetes. J. Biol. Chem..

[35] Da Silva F., Jian Motamedi F., Weerasinghe Arachchige L.C., Tison A., Bradford S.T., Lefebvre J., Dolle P., Ghyselinck N.B., Wagner K.D., Schedl A. (2021). Retinoic acid signaling is directly activated in cardiomyocytes and protects mouse hearts from apoptosis after myocardial infarction. Elife.

[36] Ghyselinck N.B., Duester G. (2019). Retinoic acid signaling pathways. Development.

[37] Yang N., Parker L.E., Yu J., Jones J.W., Liu T., Papanicolaou K.N., Talbot C.C., Margulies K.B., O’Rourke B., Kane M.A., Foster D.B. (2021). Cardiac retinoic acid levels decline in heart failure. JCI Insight.

[38] Yamaguchi A., Botta E., Holinstat M. (2022). Eicosanoids in inflammation in the blood and the vessel. Front. Pharmacol..

[39] Chawengsub Y., Gauthier K.M., Campbell W.B. (2009). Role of arachidonic acid lipoxygenase metabolites in the regulation of vascular tone. Am. J. Physiol. Heart Circ. Physiol..

[40] Palmu J., Watrous J.D., Mercader K., Havulinna A.S., Lagerborg K.A., Salosensaari A., Inouye M., Larson M.G., Rong J., Vasan R.S. (2020). Eicosanoid inflammatory mediators are robustly associated with blood pressure in the general population. J. Am. Heart Assoc..

[41] Wynne-Edwards K.E., Edwards H.E., Hancock T.M. (2013). The human fetus preferentially secretes corticosterone, rather than cortisol, in response to intra-partum stressors. PLoS One.

[42] Casey M.L., Howell M.L., Winkel C.A., Simpson E.R., MacDonald P.C. (1981). Deoxycorticosterone sulfate biosynthesis in human fetal kidney. J. Clin. Endocrinol. Metab..

[43] Berghuis P., Rajnicek A.M., Morozov Y.M., Ross R.A., Mulder J., Urbán G.M., Monory K., Marsicano G., Matteoli M., Canty A. (2007). Hardwiring the brain: endocannabinoids shape neuronal connectivity. Science.

[44] Alpár A., Tortoriello G., Calvigioni D., Niphakis M.J., Milenkovic I., Bakker J., Cameron G.A., Hanics J., Morris C.V., Fuzik J. (2014). Endocannabinoids modulate cortical development by configuring Slit2/Robo1 signalling. Nat. Commun..

[45] Kawasaki H., Hori T., Nakajima M., Takeshita K. (1988). Plasma levels of pipecolic acid in patients with chronic liver disease. Hepatology.

[46] Mihalik S.J., Moser H.W., Watkins P.A., Danks D.M., Poulos A., Rhead W.J. (1989). Peroxisomal L-pipecolic acid oxidation is deficient in liver from Zellweger syndrome patients. Pediatr. Res..

[47] Tollenaar L.S.A., Lopriore E., Faiola S., Lanna M., Stirnemann J., Ville Y., Lewi L., Devlieger R., Weingertner A.S., Favre R. (2020). Post-Laser Twin Anemia Polycythemia Sequence: Diagnosis, Management, and Outcome in an International Cohort of 164 Cases. J. Clin. Med..

[48] Covarrubias A.J., Perrone R., Grozio A., Verdin E. (2021). NAD+ metabolism and its roles in cellular processes during ageing. Nat. Rev. Mol. Cell Biol..

[49] Delanty N., Reilly M., Pratico D., FitzGerald D.J., Lawson J.A., FitzGerald G.A. (1996). 8-Epi PGF2 alpha: specific analysis of an isoeicosanoid as an index of oxidant stress in vivo. Br. J. Clin. Pharmacol..

[50] Patrono C., FitzGerald G.A. (1997). Isoprostanes: potential markers of oxidant stress in atherothrombotic disease. Arterioscler. Thromb. Vasc. Biol..

[51] Aizawa T., Ishizaka N., Usui S.-I., Ohashi N., Ohno M., Nagai R. (2002). Angiotensin II and catecholamines increase plasma levels of 8-epi-prostaglandin F(2alpha) with different pressor dependencies in rats. Hypertension.

[52] Taegtmeyer H., Sen S., Vela D. (2010). Return to the fetal gene program: a suggested metabolic link to gene expression in the heart. Ann. N. Y. Acad. Sci..

[53] Zhou B., Tian R. (2018). Mitochondrial dysfunction in pathophysiology of heart failure. J. Clin. Invest..

[54] Taegtmeyer H., Peterson M.B., Ragavan V.V., Ferguson A.G., Lesch M. (1977). De novo alanine synthesis in isolated oxygen-deprived rabbit myocardium. J. Biol. Chem..

[55] Havel R.J., Watkins E. (1950). The metabolism of lactate and pyruvate in children with congenital heart disease. Circulation.

[56] Greene N.M., Talner N.S. (1964). Blood lactate, pyruvate and lactate-pyruvate ratios in congenital heart disease. N. Engl. J. Med..

[57] Patgiri A., Skinner O.S., Miyazaki Y., Schleifer G., Marutani E., Shah H., Sharma R., Goodman R.P., To T.-L., Robert Bao X. (2020). An engineered enzyme that targets circulating lactate to alleviate intracellular NADH:NAD+ imbalance. Nat. Biotechnol..

[58] Williamson D.H., Lund P., Krebs H.A. (1967). The redox state of free nicotinamide-adenine dinucleotide in the cytoplasm and mitochondria of rat liver. Biochem. J..

[59] Taegtmeyer H., Lam T., Davogustto G. (2016). Cardiac metabolism in perspective. Compr. Physiol..

[60] Hall C. (2004). Essential biochemistry and physiology of (NT-pro)BNP. Eur. J. Heart Fail..

[61] Lopaschuk G.D., Collins-Nakai R.L., Itoi T. (1992). Developmental changes in energy substrate use by the heart. Cardiovasc. Res..

[62] Mick E., Titov D.V., Skinner O.S., Sharma R., Jourdain A.A., Mootha V.K. (2020). Distinct mitochondrial defects trigger the integrated stress response depending on the metabolic state of the cell. Elife.

[63] Harding H.P., Zhang Y., Zeng H., Novoa I., Lu P.D., Calfon M., Sadri N., Yun C., Popko B., Paules R. (2003). An integrated stress response regulates amino acid metabolism and resistance to oxidative stress. Mol. Cell.

[64] Xu X., Jin K., Bais A.S., Zhu W., Yagi H., Feinstein T.N., Nguyen P.K., Criscione J.D., Liu X., Beutner G. (2022). Uncompensated mitochondrial oxidative stress underlies heart failure in an iPSC-derived model of congenital heart disease. Cell Stem Cell.

[65] Orczyk-Pawilowicz M., Jawien E., Deja S., Hirnle L., Zabek A., Mlynarz P. (2016). Metabolomics of Human Amniotic Fluid and Maternal Plasma during Normal Pregnancy. PLoS One.

[66] Menon R., Jones J., Gunst P.R., Kacerovsky M., Fortunato S.J., Saade G.R., Basraon S. (2014). Amniotic fluid metabolomic analysis in spontaneous preterm birth. Reprod. Sci..

[67] Romero R., Mazaki-Tovi S., Vaisbuch E., Kusanovic J.P., Chaiworapongsa T., Gomez R., Nien J.K., Yoon B.H., Mazor M., Luo J. (2010). Metabolomics in premature labor: a novel approach to identify patients at risk for preterm delivery. J. Matern. Fetal Neonatal Med..

[68] Baraldi E., Giordano G., Stocchero M., Moschino L., Zaramella P., Tran M.R., Carraro S., Romero R., Gervasi M.T. (2016). Untargeted metabolomic analysis of amniotic fluid in the prediction of preterm delivery and bronchopulmonary dysplasia. PLoS One.

[69] Hallingström M., Barman M., Savolainen O., Viklund F., Kacerovsky M., Brunius C., Jacobsson B. (2022). Metabolomic profiles of mid-trimester amniotic fluid are not associated with subsequent spontaneous preterm delivery or gestational duration at delivery. J. Matern. Fetal Neonatal Med..

[70] Dunn W.B., Allwood J.W., Van Mieghem T., Morris R.K., Mackie F.L., Fox C.E., Kilby M.D. (2016). Carbohydrate and fatty acid perturbations in the amniotic fluid of the recipient twin of pregnancies complicated by twin-twin transfusion syndrome in relation to treatment and fetal cardiovascular risk. Placenta.

[71] Van Mieghem T., Doné E., Gucciardo L., Klaritsch P., Allegaert K., Van Bree R., Lewi L., Deprest J. (2010). Amniotic fluid markers of fetal cardiac dysfunction in twin-to-twin transfusion syndrome. Am. J. Obstet. Gynecol..

[72] Guilherme R., Patrier S., Gubler M.C., Lemercier D., Guimiot F., Dommergues M. (2009). Very early twin-to-twin transfusion syndrome and discordant activation of the renin-angiotensin system. Placenta.

[73] Mahieu-Caputo D., Meulemans A., Martinovic J., Gubler M.-C., Delezoide A.-L., Muller F., Madelenat P., Fisk N.M., Dommergues M. (2005). Paradoxic activation of the renin-angiotensin system in twin-twin transfusion syndrome: an explanation for cardiovascular disturbances in the recipient. Pediatr. Res..

[74] Gitlin D., Kumate J., Morales C., Noriega L., Arévalo N. (1972). The turnover of amniotic fluid protein in the human conceptus. Am. J. Obstet. Gynecol..

[75] Ritchie M.E., Phipson B., Wu D., Hu Y., Law C.W., Shi W., Smyth G.K. (2015). limma powers differential expression analyses for RNA-sequencing and microarray studies. Nucleic Acids Res..

[76] Chen Q., Kirk K., Shurubor Y.I., Zhao D., Arreguin A.J., Shahi I., Valsecchi F., Primiano G., Calder E.L., Carelli V. (2018). Rewiring of Glutamine Metabolism Is a Bioenergetic Adaptation of Human Cells with Mitochondrial DNA Mutations. Cell Metab..

[77] Sud M., Fahy E., Cotter D., Azam K., Vadivelu I., Burant C., Edison A., Fiehn O., Higashi R., Nair K.S. (2016). Metabolomics Workbench: An international repository for metabolomics data and metadata, metabolite standards, protocols, tutorials and training, and analysis tools. Nucleic Acids Res..

