# Supplementary material for: Fetal metabolic adaptations to cardiovascular stress in twin-twin transfusion syndrome
Source: iScience. 2023 Jul 20;26(8):107424. doi: 10.1016/j.isci.2023.107424 (PMC10415929; doi:10.1016/j.isci.2023.107424)
Supplement: Document S1. Figures S1–S3 and Tables S1, S3, and S5 [file mmc1.pdf]

iScience, Volume *26*

Supplemental information

Fetal metabolic adaptations to cardiovascular stress in twin-twin transfusion syndrome

Jacqueline G. Parchem, Huihui Fan, Lovepreet K. Mann, Qiuying Chen, Jong H. Won, Steven S. Gross, Zhongming Zhao, Heinrich Taegtmeyer, and Ramesha Papanna

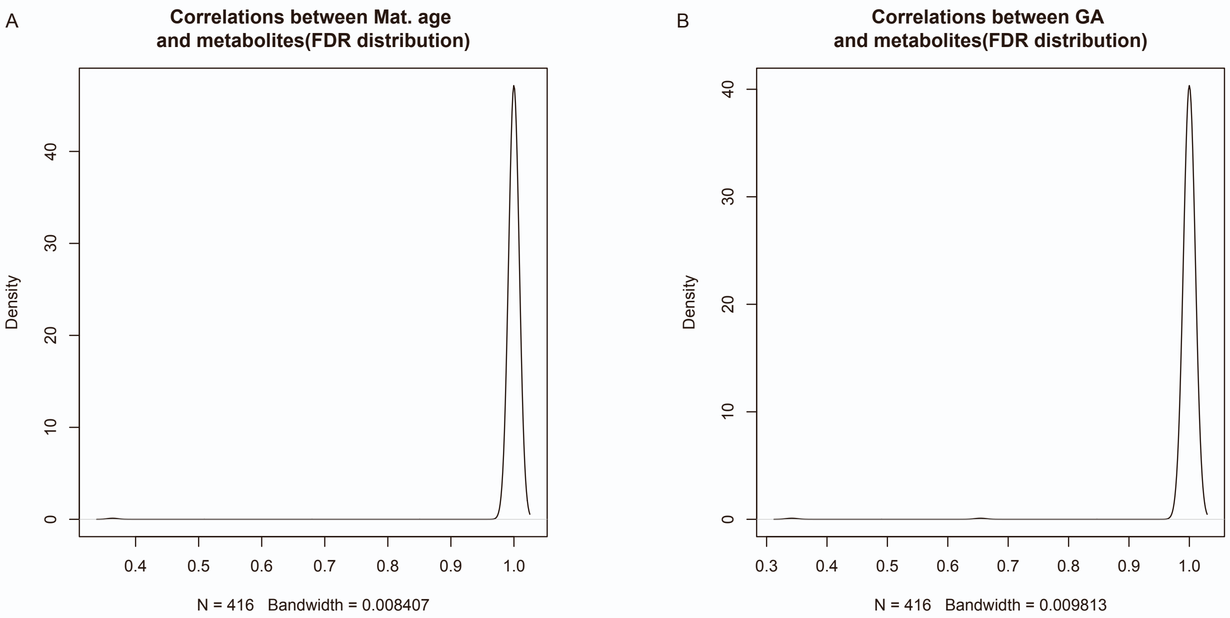

Figure S1. Distribution of the correlation significance between covariates and metabolite profile. Related to Table 1 and Figure 1. Maternal age and gestational age had no impact on metabolite profiles.

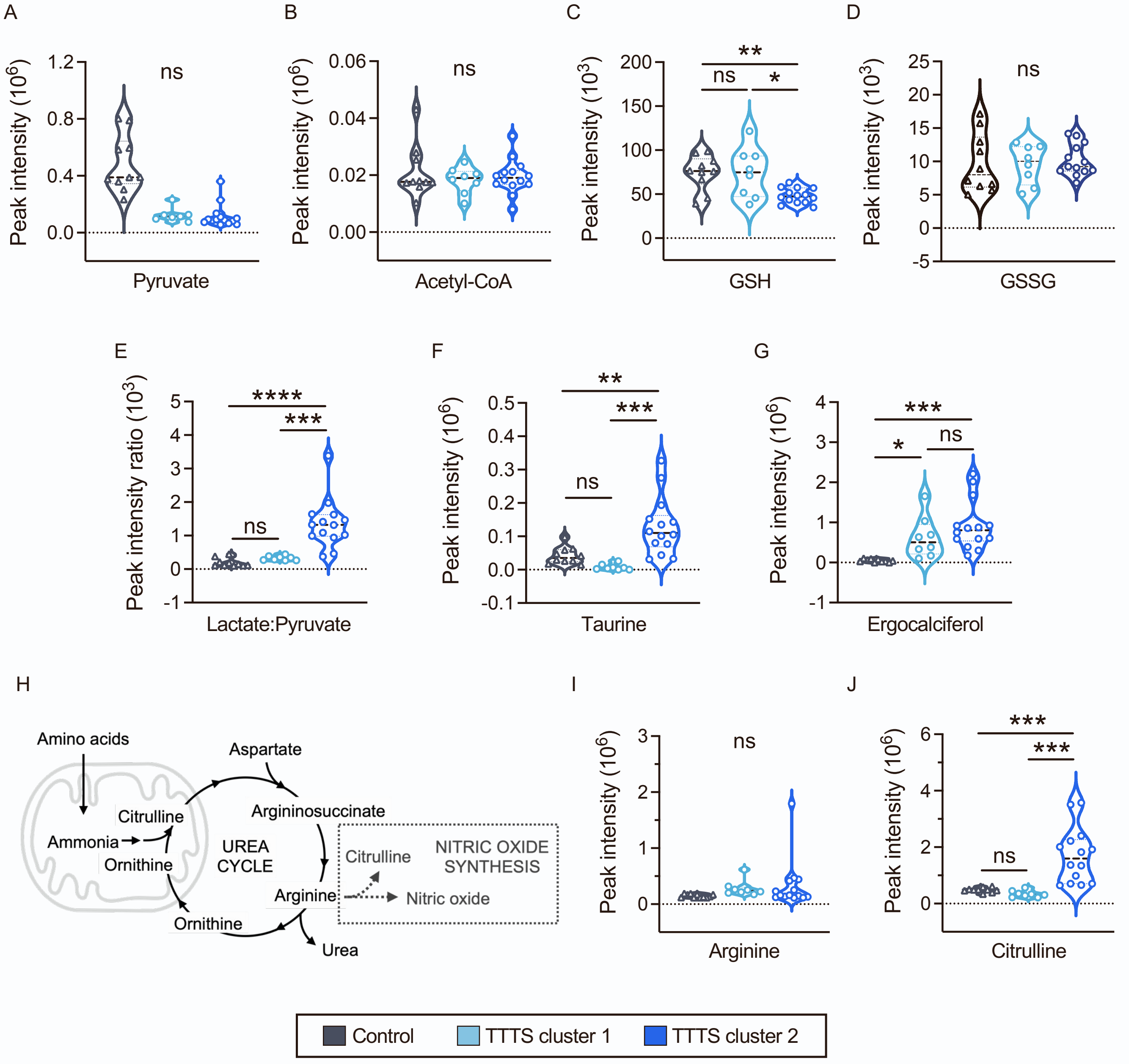

Figure S2. Distinct clusters of TTTS are defined by glycolytic metabolites, redox stress, and altered nitrogen metabolism. Related to Figure 2. Normalized mass spectrometry peak intensities for amniotic fluid metabolites in control, TTTS cluster 1, and TTTS cluster 2. (A-B) Similar levels of glycolytic intermediates, pyruvate and acetyl-CoA. (C-E) Markers of redox state, glutathione (GSH), oxidized glutathione (GSSG), and lactate-to-pyruvate ratio suggest redox imbalance in TTTS cluster 2. (F-G) Levels of antioxidants, taurine and ergocalciferol. (H). Schematic of the urea cycle and the production of citrulline and nitric oxide from arginine. (I-J) Similar arginine and increased citrulline in TTTS cluster 2. One-way ANOVA with Tukey's multiple comparisons test. ns, not significant, *$p \leq 0.05$, **$p \leq 0.01$, ***$p \leq 0.001$, ****$p \leq 0.0001$.

A

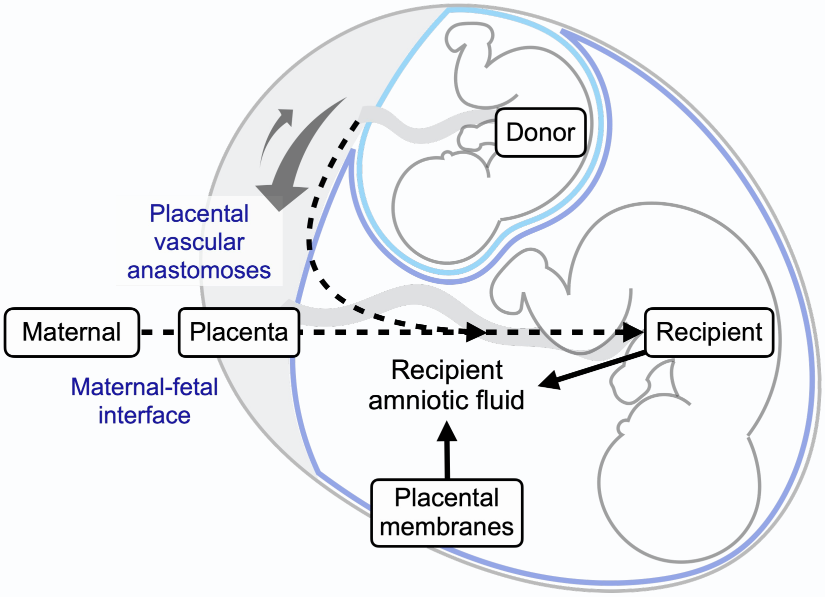

Figure S3. Schematic of direct and indirect contributors to recipient amniotic fluid metabolites. Related to Figure 1.

SUPPLEMENTAL TABLES

Table S1. Clinical characteristics of TTTS and control pregnancies, related to Table 1.

Group	Study ID	Maternal age, years	Gestational age, weeks	Fetal sex, female	Indication for amniocentesis or TTTS stage
Control	C1	32	16	0	Sickle cell disease
	C2	31	17.3	1	Abnormal screening
	C3	44	16.0	1	Maternal age
	C4	37	22.1	1	Maternal age
	C5	31	20.3	0	Abnormal screening
	C6	30	16.0	0	Prior pregnancy with genetic condition
	C7	32	17.1	.	Prior pregnancy with genetic condition
	C8	28	21	1	Abnormal screening
	C9	39	17.1	1	Abnormal screening
	C10	33	18.4	0	Sickle cell disease
TTTS	T1	31	21.1	0	3D
	T2	28	21.3	0	3D
	T3	21	20.7	1	3DR
	T4	34	20.7	1	3R
	T5	21	18.9	1	3R
	T6	40	16.4	1	3DR
	T7	30	21.4	0	3DR
	T8	27	20.4	1	3R
	T9	23	18.3	0	3D
	T10	18	19.7	0	3R
	T11	33	21.0	1	3R
	T12	31	17.9	0	3R
	T13	31	21.6	0	3R
	T14	20	19.3	1	3R
	T15	21	19.7	0	3D
	T16	27	17.6	1	3D
	T17	25	20.4	1	3D
	T18	28	17.0	0	3D
	T19	27	21.7	1	3R
	T20	25	16.7	1	3R
	T21	26	18.6	1	3R
	T22	21	20.6	0	3D

TTTS, twin-twin transfusion syndrome; D, donor; R, recipient. Abnormal screening defined as increased risk based on genetic screening results or non-structural ultrasound findings. 1 = yes; 2 = no.

Table S3. Clinical characteristics of TTTS clusters, related to Table 2.

Cluster	ID	Age, years	BMI, kg/m^2	GA, weeks	Fetal sex, F	Recipient					Donor			Weight disc., %	TAPS
						MVP, cm	MR/TR	UA	DV	UV	UA	DV	UV		
1	T3	21	25.7	20.7	1	8.8	0	0	2	1	1	1	0	28.7	1
	T4	34	26.2	20.7	1	13.0	0	0	2	0	0	.	1	24.6	0
	T6	40	30.0	16.4	1	8.2	0	0	1	0	1	1	0	38.9	0
	T13	31	23.0	21.6	0	11.9	0	1	0	1	0	0	0	11.0	0
	T14	20	34.7	19.3	1	9.0	0	0	0	1	0	0	0	9.9	0
	T15	21	26.8	19.7	0	9.8	0	0	0	0	1	0	0	24.6	0
	T16	27	25.6	17.6	1	7.3	0	0	0	0	1	0	0	29.2	0
	T22	21	28.2	20.6	0	15	0	0	1	0	1	0	0	40.2	0
2	T1	31	25.5	21.1	0	13.9	1	0	0	0	1	0	0	22.8	0
	T2	28	24.1	21.3	0	11.3	0	0	1	0	1	0	1	36.8	0
	T5	21	28.3	18.9	1	11.2	0	1	2	1	0	.	1	3.6	0
	T7	30	27.1	21.4	0	11.7	0	1	0	1	1	0	0	5.6	0
	T8	27	35.3	20.4	1	13.1	1	0	2	1	0	0	0	25.2	0
	T9	23	21.8	18.3	0	6.4	0	0	2	0	1	1	1	47.0	0
	T10	18	23.9	19.7	0	10.4	.	0	1	1	0	0	0	12.1	0
	T11	33	38.6	21.0	1	13.0	0	1	0	1	0	0	0	12.6	1
	T12	31	54.2	17.9	0	8.3	0	0	2	0	1	0	0	48.5	0
	T17	25	18.9	20.4	1	14.2	0	0	0	0	1	0	0	36.8	0
	T18	28	20.4	17.0	0	8.6	1	0	1	0	1	0	0	19.6	0
	T19	27	35.1	21.7	1	14.1	.	1	1	1	1	0	0	6.8	0
	T20	25	19.7	16.7	1	9.6	0	0	1	1	0	1	0	9.8	0
	T21	26	24.3	18.6	1	16.6	0	0	1	1	0	0	0	32.9	0

BMI, body mass index; GA, gestational age; F, female; MVP, maximum vertical pocket; MR/TR, mitral/tricuspid regurgitation; UA, umbilical artery; DV, ductus venosus; UV, umbilical vein; disc, discordance; TAPS, twin anemia-polycythemia sequence. 1 = yes/present; 0 = no/absent. For Doppler studies: 0 = normal; 1 = absent end-diastolic flow (UA), absent a-wave flow (DV), or pulsatile flow (UV); 2 = reversed end-diastolic flow (UA) or reversed a-wave flow (DV).

Table S5. Outcomes of TTTS cases according to metabolite profile, related to Figure 3.

Outcome	Cluster 1 (n=8)	Cluster 2 (n=14)	*P*
Perinatal death (fetal or neonatal)			
Pregnancies affected	3 (37.5)	2 (14.3)	0.31
Fetus or neonate	4/16 (25)	2/28 (7.1)	0.17
Preterm birth <34 weeks	4 (50)	6 (43.9)	>0.99
Preterm premature rupture of membranes	2 (25.0)	4 (28.6)	>0.99
Birth GA, weeks	33.5 (22.3-36.6)	34.6 (25.6-36.4)	0.90
Interval, laser to delivery, d	88.0 (26.3-120.3)	97.5 (31.0-127.3)	0.91

Data are n (%) or median (IQR). Fisher's exact test or Mann-Whitney test.
